# Analysis of the dental care queries in the “Mouth-Nose” discussion forum of the French association of patients with Gougerot-Sjögren’s syndromes and dryness

**DOI:** 10.1186/s12903-022-02450-5

**Published:** 2022-09-22

**Authors:** P. Danvers, J. Saide, F. Decup, R. Seror, R. Belkhir, M. Gosset

**Affiliations:** 1 Université Paris Cité, Faculté de Santé, Odontologie, 92120 Montrouge, France; 2 Association Française Pour Les Patients Atteints de Gougerot Sjögren Et Des Syndromes Secs, 75018 Paris, France; 3 Service de Médecine Bucco-Dentaire, AP-HP, Hôpital Charles Foix, 94200 Ivry/seine, France; 4 Laboratory of Orofacial Pathologies, Imaging and Biotherapies URP2496, Université Paris Cité, Faculté de Santé Odontologie, 1 rue Maurice Arnoux, 92120 Montrouge, France; 5 Department of Rheumatology, Université Paris-Saclay, Assistance Publique-Hôpitaux de Paris (AP-HP), CHU Bicêtre, Le Kremlin-Bicêtre, France

**Keywords:** Sjögren syndrome, Sicca, Online forum, Oral care, Dentist

## Abstract

**Background:**

Primary Sjögren Syndrome is a rare autoimmune systemic disease characterized by impaired secretory functions of the exocrine gland. One of the main clinical features is dry mouth and subsequent oral diseases, which are also found in patients with Sicca. This leads to a marked deterioration in the quality of life and the patient’s search for information and solutions. Many patients turn to patients’ associations that offer moments of sharing to their members, especially through online discussion forums. Today, these forums represent quality material for a sociological or biomedical analysis of patients' concerns, as close as possible to their daily lives. Our objective is to analyze the concerns of patients with SS or Sicca regarding their dry mouth especially dental care.

**Methods:**

In this cross-sectional observation study, a quantitative analysis of the Mouth-Nose online forum discussion of the French Association of Patients with Gougerot-Sjögren’s Syndromes and Dryness have been performed. After reading and re-reading, initial request themes, topics, and subtopics were established and coding was performed. Then, the 885 threads were classified depending the initial request, pragma-linguistic indices and the main topic discussed in the thread. After identifying the threads dealing with dental care, we looked at which types of care were most discussed and classified the discussions according to whether or not the patient was satisfied with their care at the dentist.

**Results:**

The majority of the initial requests are posts for experiences sharing and/or advice. The topic of “dental care” is one of the main concerns of the forum users. Among the threads that concern dental care, requests to share experience with implants are in the majority. Finally, the majority of the posts on dental care relate to care in private dental practice, deals with dental implants and prevention and resulted mainly in patient satisfaction.

**Conclusions:**

Analysis of the forum reveals importance of patient concerns about prevention, and care costs due to implant treatment, which add to disease burden. Most of messages relate favorable experiences with their dentists, which is in line with the approach of sharing experiences and support characteristic of a forum.

## Background

Primary Sjögren Syndrome (pSS) is a rare (0.1–1/10000) autoimmune systemic disease characterized by impaired secretory functions of the exocrine [1, 2]. pSS is characterized by association of a symptomatic triad (dryness, pain and fatigue) with various systemic manifestations. While systemic involvement affects about 30% of patients, sicca, mainly ocular and oral, is present in almost all patients, and cause high disease burden and a marked deterioration in quality of life. When associated with other systemic autoimmune diseases (such as rheumatoid arthritis, systemic lupus erythematosus, or systemic sclerosis), Sjögren’s syndrome is called “associated” rather than “secondary”. Finally, a dry syndrome can exist without a diagnosis of Sjogren’s syndrome. Sicca syndrome is thought to affect between 1 and 46% of the population with an increase in prevalence with age and gender [3]. Potential causes of dry mouth syndrome are numerous (e.g. salivary glands irradiation, certain medications). Dry mouth is associated with an increased risk of oral diseases (candidiasis, dental caries, dental erosions) [4] and a lower quality of oral life [5]. Dentist thus has a key role to play in detection of Sicca syndrome and pSS and in implementation of an adapted prevention of oral risks in these patients.

The impact of pSS on patients’ quality of life has been explored in both quantitative and qualitative studies. In quantitative studies, SF-36 (The short Form Health Survey in 36 item) questionnaire assessing general health-related quality of life and the OHIP-14 or -49 (Oral Health Impact Profile in 14- or 49-item) assessing oral health-related quality of life were used to allow statistical comparison between groups with Sicca or pSS compared to a control group. These studies show a deterioration in health-related quality of life in patients with dry mouth, characterized by a reduction in almost all domains of SF-36 [6, 7], and increase in the OHIP scores [8]. Qualitative studies provide additional information by exploring the lived experience of patients with pSS. They aimed to understand the experience and daily life of patients with pSS, including the emotional burden and beliefs associated with the disease. These qualitative studies highlighted physical consequences of dry mouth as already described in the literature (difficulty moving the lips and mouth, partial or total loss of taste and smell, difficulty eating, swallowing and speaking). Unfortunately, impact of dry mouth on quality of life of patients with pSS could not be explored, authors concluded that disease must be considered as a whole and that its symptoms cannot be isolated from each other in patients’ speech [9, 10].

Online support forums are also defined as online health communities or online support groups. They correspond to online services with features that enable members to communicate with each other. They can play an important role in helping and supporting individuals with self-care and self-management of various health-related challenges and are highly investigated in medical researches (e.g. [11]). Today, online patient discussion forums represent a material of quality for a sociological or biomedical analysis of patients’ concerns, as close as possible to their daily life. French Association of Patients with Gougerot-Sjögren’s Syndromes and Dryness (AFGS) gathers in France patients with dry mouth or Sjögren’s syndrome, mainly affected by a primary syndrome. AFGS has proposed for several years a forum of discussion “Dry mouth” for their member patients. Thus, thanks to AFGS collaboration, we carried out an analysis of problems discussed by patients, and structured them by fields in order to better understand difficulties and questions of patients with dry mouth in their daily life and how the dentist is involved in patient with SS or Sicca oral care.

## Methods

### Design of the study

This study is a cross-sectional observation with quantitative content analysis of the Mouth-Nose online forum discussion of the French Association of Patients with Gougerot-Sjögren's Syndromes and Dryness (AFGS).

### Data retrieval

Data that were used for our retrospective analysis correspond to 885 messages sent between March 2006 and February 2020, by AFGS members on discussion forum entitled “Mouth-Nose”, available on association’s website. Anonymous data have been extracted by AFGS in an Excel^®^ file. The “participatory framework” of “Mouth-Nose” forum includes all AFGS members who have at any time consulted or participated in the forum. Note that all participants of this forum are AFGS members, up to date in their membership fees.

### Data analysis

#### Identification and classification of threads depending on initial request

Data analysis was carried out according to the methodology developed by Matta et al. [12]. Briefly, after several data readings, we first isolated forum threads. A thread includes the initial request (first post) as well as all responses to that request. Number of messages of each thread has been counted. 188 threads were identified, two of which were excluded because they corresponded to two posts from the same person and on the same day, without giving rise to an exchange, and without any mention of Sjögren’ syndrome or Sicca. Then, each of the 186 threads was classified into one of the initial request categories proposed by Matta et al. [12] (*Initial request *1–4*:* Experience sharing, Advice, Emotional support, Informational request) or that we created (*Initial request *5: Testimonials).

Sometimes two main requests were identified in the first thread message. Therefore, we proposed to group some initial queries as follows (*Initial Request *6*:* Experience Sharing and Advice; *Initial Request *7** = **Experience Sharing and Informational request; *Initial Request *8 = Informational request and Advice).

#### Identification and classification of threads based on pragma-linguistic indices

Threads were analyzed to identify pragma-linguistic cues according to five categories proposed by Nada Matta et al. [12]. We also proposed six other pragma-linguistic cues to analyze in the where dentist was mentioned, dentist role in management of patients with pSS or Sicca 5 Table 1).

**Table 1:** List and examples of the pragma-linguistic indices found in the Mouth-Nose forum threads

Pragma linguistic indices	Number	Name	Examples
*In all the threads*	1	Encouragement/praise	“*I wish you a lot of courage*”
	2	Criticism/disagreement	“*The chewing-gum is not recommended because it is a lure for the stomach*”* (response to a user who explained that chewing-gum relieved her oral dryness*)
	3	Advice/information	“*I advise to go to the site documentation database, in the services tab*”
	4	Assessment of a situation and request for additional information	“*Many of you are talking about oral gel but what kind of oral gel is it?*”
	5	Assessment of an expertise and experience sharing	“*I also had side effects: sweating and chills after taking this drug against hyposalivation*”
*Specific to dental cares*	6	Request for advice	“*I consulted at the dental department in the hospital where pSS is known*”
	7	Solution or advice provided by a dentist in a private dental office	“*My dentist made me gutters that I fill with fluoride *2* or *3* times a week, which relieves me of extreme teeth sensitivity*”
	8	Solution or advice provided by a dentist in a hospital	“*I learned how to brush my teeth at the dental department of Brest; I use brushes and dental floss, as well as toothpaste and special dry mouth mouthwash (without alcohol)*”
	9	Solution or advice not provided by dentist	“*I run to a dentist, a stomatologist, but no one can improve my pain. I no longer know where to go*”
	10	Satisfaction with dental care	“*Thanks to treatment prescribed by my dentist, everything is over, I no longer have any pain*”
	11	Dissatisfaction with dental care	“*I have seen my dentist several times about this problem but there is nothing he can do*”

#### Identification of main topics threads

An iterative reading of 186 threads allowed us to identify 8 main topics (Dental Care; Dryness; Oral dryness treatments; Sensory and functional alteration; Other oral clinical symptoms; Parotid and ear pain; Inflammation of mucosal membranes; Ohers) addressed in threads. For the “Dental care” topic, 4 subtopics were identified: Prevention; Soft tissue treatments; Dental implants; Other treatments.

#### Identification of main topics threads on dental care experiences

The reading of the 186 threads identified 34 threads about patients’ experiences with dentist. These threads were analyzed to identify: dentists’ practice location (hospital or private office); dentists’care (number and nature); whether dentists addressed patient's concerns (Yes/No); and the satisfaction with the care received (Yes/No). Note that in absence of a solution, we systematically considered that there was a dissatisfaction with dental care.

Dentist's care have been classified in seven categories (Table 2).

**Table 2:** List and examples of the Dental Care experiences reported in the Mouth-Nose forum threads

Number	Category	Definition
1	Health education	Explanation of the link between SS and oral health
2	Prevention	Oral hygiene materials and techniques, frequency of dental visits, fluoride trays
3	Soft tissue treatment	Mouth ulcers, mycosis, dry lips, gingivitis
4	Dental tissue treatments	tooth decays, dentinal hypersensitivity
5	Oral rehabilitation with dental implants	Placement of implants, bone grafting, peri-implantitis
6	Salivary glands pathologies	Parotid glands or sublingual glands stones, parotiditis
7	Others	Prosthetic equilibration, infectious complications

### Ethical considerations

This study is a neutral observation of discussions strictly carried out in an online space without any interaction with the members of the community who participated in the forum Mouth-Nose of AFGS. The forum was analyzed with a high level of confidentiality and anonymity preservation [13]. If thread data presented information allowing people identification, this one was not analyzed (no thread concerned). AFGS, in accordance with the European Regulation (EU) 2016/679 of the European Parliament and of the Council of 27 April 2016 on the protection of individuals with regard to the processing of personal data and on the free movement of such data, applied in France (General Data Protection Regulation), does not keep information relative to people who posted messages. Consequently, no informed consent was required, according to [14].

Favorable approval from the Ethics Committee of CERAPHP Center was obtained on October 20, 22 (IRB registration #00,011,928).

## Results

### Characteristics of the forum

From 2006 (opening of the forum) to 2020 (data collection), 188 initial requests were posted. Between 2006 and 2020, the forum was mostly active between 2010 and 2017 (from 13 to 18 posts per year). 153 of the 186 initial posts (82.3%) give rise to answers from the community, highlighting that forum discussion is active. Conversely, some threads consist of only one message, i.e. the initial post. Thus, 33 of the 186 threads (17.7%) did not lead to a sequence of messages, and are named “truncated messages”. We did not observe any correlation between truncated messages and a particular topic, as a large variety of topics (e.g. in the “Experiential sharing topic”: Sub-Topic 1.3: dental implants and Sub-Topic 1.4: other treatments) did not lead into a discussion.

### Experience sharing, for help and support, is the main request of the threads in the forum

Table 3 reveals that 74.18% of AFGS members participate in the forum to share their experience or to seek advice, or both. In their discussions, they mainly seek for solutions and remedies to reduce dryness symptoms intensity and thus improve daily life. They also seek to be comforted in their choice of treatment, product purchases or simply in the onset of their symptoms. At the opposite, we note that the part of requests for strict emotional support (2.15%) is rare and that they are mostly requests for comfort. Some testimonials (9.13% of the initial requests) illustrate the willingness of some Internet users to spontaneously share their experience concerning treatments that have worked for them in the hope that they can help other patients, and without making a request. Finally, the last two categories are associations of information requests with a request for experience sharing notably on the appearance of their symptoms (3.2%), or associations of information requests with requests for advice (0.5%).

**Table 3:** Distribution of initial requests

Initial request	n = (%)
1. Experience sharing e.g. “*Has anyone experienced the same thing? if so, could they share their experience with me?*”	70 (37.63)
2. Advice e.g. “*Does anyone have a treatment to reduce my dryness at night?*”	35 (18.81)
3. Emotional support e.g. “*Help me, I don’t know what to do to improve my dryness*”	4 (2.15)
4. Informational request e.g. “*Does anyone have any information on the new product available?*”	20 (10.75)
5. Testimonials e.g. “*For mouth ulcers, I use a trick given by a stomatologist: […] perhaps that could relieve you too*”	17 (9.13)
6. Experience sharing and advice e.g. “*[…] I would like to know if anyone has had this problem. […] If someone could help me, […]*”	33 (17.74)
7. Experience sharing and informational request e.g. “*I would like know if having bitter mouth is a one of pSS symptoms? (…) If someone has lived and overcome this, let him answer me*”	6 (3.22)
8. Informational request and advice	1 (0.53)

The 186 threads were classified into one of eight initial request categories proposed by Matta et al. [12] (*Initial request *1–4) or proposed by our group (*Initial request *5–8)

Interestingly, 10.75% of initial requests correspond to a search for information about dental products or dental consequences of the condition. In majority of cases, they seek either to confirm or to complete information provided by their peers or by dentists and which they consider unsatisfactory or anxiety-provoking (on the evolution of their health condition, certain treatments or administrative procedures). For example, Ingrid was afraid of losing her teeth: “*Hello, (…) I'm a little afraid to ask the question: do we really lose our teeth necessarily even if we go to the dentist or are there solutions to prevent this?? I was told about a fluorine tray: does it work? Thank you for answering me because it really scares me to lose my teeth (I’m only *33* years old and I love my little smile !!)* and Alix needs information on dental implant procedures*:* “*Hello, probably suffering from a sicca syndrome, I am considering having implants fitted. Are there any risks, contraindications? How to get a referral from a dentist or stomatologist expert in this pathology? Thank you very much for lighting my lantern*”*.*

Main pragma-linguistic indices of messages are related to the categories “Advice/information” and/or “Experience sharing” with the aim to share experience on treatments, and tips to reduce symptoms intensity and improve quality of life. We also noticed a lot of “encouragement/compliments” messages, and on the contrary, very few “criticisms and agreements”, which reveals the importance of the values of mutual aid and solidarity. A lot of gratitude and moral support emerge from these exchanges. Indeed, patients seem so overwhelmed by the degenerative and incurable aspect of the disease that any experience with a new treatment is of great help to them.

### Dental care is the main topic discussed in the forum

The analysis of topics addressed in initial queries allows us to see that “Dental care” query is the main one addressed by users of Mouth Nose forum (21.5%) (Table 4). These interventions relate mainly to questions about prevention as in Chantal’s post “*Hello, I have a simple question: is the use of an electric toothbrush advisable or not in our case of dry mouth and fragility, if someone could give me an opinion? Thank you and good day*!» and soft tissue treatments, notably around dental implants “e.g. *I have had implants for *5* years and the gums retract a lot leaving them too exposed. My dentist has been insisting for *2* years to do a gum transplant to me, which I oppose thinking that it will not work because of lack of saliva and also because we make too many autoantibodies. […] Has anyone ever needed this transplant? Courage to all. Arlette*”.

**Table 4:** Distribution of threads topics

Topics	n = (%)
1. Dental care	40 (21.50)
2. Dryness	36 (18.82)
3. Oral dryness treatments	27 (14.52)
4. Sensory and functional alteration	29 (15.60)
5. Other oral clinical symptoms	19 (10.22)
6. Parotid and ear pain	8 (4.3)
7. Inflammation of mucosal membranes	8 (4.3)
8. Others	20 (10.75)

After reading, the 186 threads were classified according to the main theme of the thread into one of the eight categories proposed

Among the posts dealing with “dental care” (Table 5), threads mainly deal with "implant treatments". Indeed, many patients with pSS suffer from partial or total edentulism and oral rehabilitation with removable prosthesis is described as difficult because of lack of saliva (which leads to traumatic irritations, candidiasis…). Therefore, most of forum users seek information about dental implants, which seem to be the solution of choice to improve speech, chewing, and oral comfort. In the majority of threads (64%), they are looking for “shared experiences” on the possibility of being treated with dental implants in view of their medical condition (“*Hello I wear an inferior denture but I don't support it because it moves and causes me injuries. My dentist proposes me the placement of implants (…) I admit that I hesitate because it is an expensive operation and I do not know if it is possible with an pSS, I ask myself a lot of questions also thank you for giving me your observations if you have implants because every meal becomes torture for me. Thanks a lot for your answers. GHS*”); the implementation protocol (“*Hello, causing by my dry mouth, I have to undergo a bone graft of lower jaw; I would like to know the different stages of this operation, thank you in advance ROLAND*”) and the financial cost of the treatment (balanced with the advantages) (“*Hello, […] My dentist advised me *4* implants for the top and the device would be attached to it. It will cost me around *9000* euros with a very low refund. I have made an exceptional request to the CPAM* [public health insurance] *and I am awaiting the answer. Have other people put implants and are they satisfied? […] Marie*”.

**Table 5:** Distribution of “Dental care” sub-topics of the threads

Dental care subtopics	n = (%)
1. Prevention	6 (15)
2. Soft tissue treatments	9 (22.5)
3. Dental implants	14 (35)
4. Other treatments	11 (27.5)

We classified the 40 threads dealing with Dental Care, according to fours dental care subtopics we identified

### Dental’s care experiences

In the 34 threads of the forum dealing with patients’ experiences with the dentist, we have counted 55 dentists ‘intervention (i.e. procedures and/or advice & prevention) reported by the internet users. 48 of the 55 surgical dentist interventions took place in a private dental practice (87.3%) against 7 (12.7%) in a hospital.

In many threads, the dentist was described as involved in informing patients about education on links between oral health and pSS (e.g. “*Hello, while reading your message I noticed that your teeth are breaking, just like me, on my side especially the front ones (incisors, bottom and top) … Having accidentally received a shock in the upper jaw there is About *13* years old, I thought that was the consequence of it but my dentist told me that it is rather related to the dry syndrome that I have for *14* years (…). Good luck, Dany*” and in educating the patient about health behaviors to follow to prevent lesions development.

In only 9.1% of the threads, it was reported that the dentist was unable to give patients some answers or solutions, as in this post “*Hello, (…) I have a big “hot–cold*”* problem on my teeth, it’s very unpleasant, because the teeth hurt a lot when eating and drinking hot or cold. I saw my dentist on several occasions regarding this subject and there is nothing he can do. I would like to know if other people have this problem? Thank you for your reply*”*.*

Interestingly*,* for 41 of the 55 dentist surgical interventions (74.5%), the patient was satisfied with care, advice or information provided by the dentist as in the Monique’s post (“*Solution from my stomatosis to relieve pain and be able to eat in peace with aphthous lesions: dynexan*^®^ 2*% oral cream.*”* What happiness! And in addition, it is reimbursed*”. Main reasons of dissatisfaction were a lack of pain relief and a low level of confidence in the health professional, who is not aware of the disease, as in the Eliane’s post “H*ello, (…) my gums have retracted and on some teeth the collar appears. He prescribed me […] toothpaste special retracted collars but I find that it dries my mouth. He knows I have a Gougerot[Sjögren]; he knew vaguely and I spoke a little more about it with him and with each visit he asks me about it. (…).*

### Prevention and treatment with dental implants are the main treatments performed by dentists discussed in the forum

Among the threads dealing with the dentist's care of AFGS forum users (Table 6), we assessed that prevention of dental, mucosal or periodontal pathologies (29.0%) and oral rehabilitation with dental implants (30.9%) were the main subtopics discussed. Oral hygiene education (equipment, technique and frequency) and frequency of check-ups were the main preventive measures discussed.

**Table 6:** Distribution of “Dental care” sub-topics of the threads

Dental care subtopics	n = (%)
1. Health education	2 (3.6)
2. Prevention	16 (29)
3. Soft tissue treatment	5 (9)
4. Dental tissue treatments	7 (12.7)
5. Oral rehabilitation with dental implants	17 (30.9)
6. Salivary gland pathologies	3 (5.5)
7. Others	2 (3.6)

Among the 186 threads, 34 threads were about patients’ experiences with dentists. These were classified into 7 subtopics according of the nature of the Dental Care discussed

## Discussion

With the known advantages and disadvantages inherent to analysis of a forum [15], AFGS Mouth and Nose forum represents a good tool to analyze patients with Sjögren and Sicca queries, as regular posts and interactions have taken place over several years. The tone in the forum threads was generally very empathetic, kind and supportive. Indeed, it has already been described that the main purpose of a patient discussion in a discussion forum is to provide support and experience sharing between individuals, which is also the case here [11, 16].

Interestingly, even if dental consequences of dryness in patients with pSS or Sicca have already been described in many studies [17, 18], we have no information on their experience directly from patient’s perspective [19] and in particular on their experiences and concerns regarding dental problems and follow-up by the dentist. In this forum, we observed that Dental care is the main subject of discussion with a lot of messages on dental implants and prevention. The interest in prosthetic treatment with dental implants for the replacement of missing teeth is obvious, mainly due to the psycho-affective impact of edentulism. This is especially true for patients with pSS or Sicca, for whom dry mouth complicates the wearing of prosthesis from a medical point of view (e.g. more oral candidiasis) and from the point of view of the patient (e.g. discomfort). However, it is an expensive treatment that many find difficult to afford. Here, this “financial stress” is also described by patients with pSS or Sicca syndrome, as already described [20].

It is also interesting to note that parotid gland alterations (i.e., pain or enlargment corresponding to parotitis), which are medical features of patients with pSS systematically investigated in their history, are less discussed than sensory and functional alterations. Therefore, this forum analysis highlights some features of dryness that are important in the daily life of the patient but remain little studied in research or in daily clinical practice. Indeed, few authors have described an alteration of taste and smell functions related to dry mouth in pSS and Sicca’s syndrome [21, 22] although they are essential components of quality of life of individuals linked to appetite, self-esteem, psychological balance and nutritional imbalance.

It was also interesting to note that patients with Sjögren’s syndrome or Sicca are mainly followed by dentists in town dental clinics rather than in hospital dental services, even in France where hospital services are specialized in rare autoimmune diseases treatment, including pSS (Centre de Référence et de Compétences). Moreover, Dentists would have played a satisfactory role in providing solutions leading for most part to patient satisfaction. However, this observation cannot be representative of reality insofar as the main objective of a discussion forum is to provide solutions between Internet users, with the common goal of living better with this disease and not to complain, which would not be constructive. Indeed, Gairy et al. reported in 2020 in a qualitative study comprised moderated online forum discussions and online one-to-one questions conducted in the USA over a 2 week period on 48 patients with pSS that the treatment needs of pSS patient are often unmet (these findings are not limited to oral care) [20].

In the study by Gairy et al. [20] most participants reported impacts on social functioning of their pSS on relationships with family and friends, leisure activities, and so on. Oral dryness consequences on a decrease in patients’ oral and general quality of life are well described. It is interesting to note that here we do not read many threads on these topics even from a very practical point of view such as difficulty swallowing or mucus in the throat. We suggest that, as described in the qualitative studies, dry mouth consequences are surely strongly interwoven with social life and cannot be distinguished from it in the patient's representations and experiences [9, 10].

Finally, the main limitation of this study is that the active population of the forum is not necessarily representative of all patients with Sicca or pSS. Indeed, the number of messages raised on the forum, with an average of 13 initial requests per year, is quite low given the number of patients with SS or Sicca in France. This could be partly explained as patients with secondary Sjogren's are more likely to join a patient association for the autoimmune disease they also have, but may also reflect a specific population active on the forum. It was open to people registered with AFGS, meaning these patients are active and invested in finding information about their disease. They could expect a moderation by a healthcare professional (whom dentists) and/or expert patients that is sometimes proposed in patients’ association forum, explaining that many queries related to dental treatment such as fluorides and dental implants. In addition, they may have already consulted other sources of information on the web, such as YouTube videos or scientific literature, without finding answers to their questions, turning to the forum for that [23, 24]. Finally, we cannot exclude that active patients on the forum are not diagnosed with the disease. In other words, this forum could be a place for a subgroup of patients who need more information from their dentists about dental care and the dental consequences of Sjögren’s syndrome.

To conclude, analysis of this online discussion forum provides insight into patients’ perspectives with pSS or Sicca’s dry mouth. It reveals importance of sensory and functional alterations in quality of life, and their concerns about prevention, and care costs which add to disease burden.

Most of messages relate favorable experiences with their dentists, which is in line with the approach of sharing experiences and support characteristic of the forums. It would be interesting to conduct qualitative studies to evaluate in depth the concerns of patients with SS, particularly in relation of the detection of their disease by the dentists, as well as the dental care.

## Data Availability

The datasets used and analysed during the current study are available on the Mouth and Nose forum of the AFGS and can be delivered from the corresponding author on reasonable request.

## Abbreviations

AFGS: French association of patients with Gougerot-Sjögren’s syndromes and dryness
OHIP: Oral health impact profile
SF-36: Short form health survey in 36 item
pSS: Primary Sjögren syndrome
